# 

**Published:** 1903-07-18

**Authors:** 


### NOTICE TO CORRESPONDENTS.

All MSS., Letters, Books for Review, and other matters intended for the Editor should be addressed The Editor, "The Hospital," The Hospital Buildings, 28 & 29 Southampton Street, Strand, W.C.

The Editor cannot undertake to return rejected MSS., even when accompanied by stamped directed envelope.

**Notice to Advertisers and Subscribers.**

All Advertisements, Orders for Copies of The Hospital, and Business Communications should be addressed to **THE MANAGER, (Not to the Editor)**, The Hospital, 28 & 29 Southampton Street, Strand, London, W.C.

The Cost of Subscription, post free, is as follows.—Per week, 3½d.; per quarter, 3s. 9d.; per half-year, 7s. 6d.; per annum, 15s. Foreign and Colonial:—Per annum, 19s. 6d., payable, in all cases, in advance.

**NOTICE.—Vol. XXXIII. of "The Hospital" (October 1902 to March 1903) in handsome cloth binding, will shortly be on sale at the office of this Journal, price 7s. 6d. Cases for binding the Numbers contained in this Volume 1s. 6d. Index to Vol. XXXIII., 2½d., post free.**

**The monthly part for July is now ready. Price 1s., by post 1s. 3d.**

### BOOKS RECEIVED.

Percy Lindley.

"Great Eastern Railway Company's Tourists' Guide to the Continent."

J. B. Lippincott Co.

"International Clinics." Thirteenth Series. Vol. I.

R. L. Sharland.

"The Scientific Roll" "Bacteria," Vol. I., No. 8.

Health Resorts Association.

"Llandrindod Wells."

George Allen.

"Jessie Vandaleur." By Ethel Colburn Mayne.

"Badmanston." By Haverfield.

John Murray.

"The Pinch of Poverty." By H. A. Vachell.

The Authors and Booksellers' Co-operative Alliance, Limited.

"Water and Air." By J. P. Sandlands, M.A.

"Quacks and What they Do." By One of Them.

"Higher Criticism as Applied to Itself." By Artemus Longsides, M.A.

"Nurses and Nursing, or How not to do It." By Sister Medicatrix.

Isbister and Co., Limited.

"The Log of the *Folly*." By Allen Upward.

Grant Richards.

"Reprobate Silver." By Roy Devereux.

"Lovely Woman." By T. W. H. Crosland.

### THE STUDENT OF MEDICINE.

### APPOINTMENTS.

**J. A. Arkwright**, M.D., District Medical Officer of the Dover Union. **J. Blumfeld**, M.B.Camb., Honorary Anæsthetist to National Dental Hospital. **Auguste Boyes**, M.B., Ch.B.Glas., Resident Medical Officer at the East Poorhouse and Hospital, Dundee. **G. H. Broinowski**, M.B.Syd., Medical Officer Narrandera Hospital, N.S.W. **Frederick S. Butler**, M.B.Melb., District Medical Officer at Beverley, West Australia. **E. N. Cunliffe**, M.B., Ch.B.Vict., Assistant Medical Officer of the West Derby Union. **John Gordon**, M.D.Melb., F.R.C.S.Eng., Surgeon to Out-patients, Melbourne Hospital. **Colin Gray**, M.B., Ch.B.Melb., Medical Officer to the Maldon Hospital and Benevolent Asylum, Victoria. **E. A. Lavery**, L.R.C.P.&S Edin., Government Medical Officer and Vaccinator at Gosford, N.S.W **Walter C. McClelland**, M.B., Ch.M.Syd., Honorary Medical Officer, Marrickville Cottage Hospital, N.S.W. **E. L. Newman**, M.B.Syd., House-Surgeon, Royal North Sydney Hospital, N.S.W. **Hugh Playfair**, M.D., F.R.C.S., Assistant Obstetric Physician to King's College Hospital. **Ward Smith**, M.B., Ch.B.Edin., F.R.C.S.Eng., Honorary Assistant Medical Officer in charge of the Electrical Department of the Bradford Royal Infirmary. **David Somerville**, M.D., D.P.H., Senior Demonstrator in Public Health, King's College, London. **W. Sproule**, M.B., M.S., Medical Officer, Burrowa Hospital, N.S.W. **Dudley C. P. Taylor**, M.R.C.S.Eng., L.R.C.P.Lond., Medical Officer and Public Vaccinator to the Sibsey District of the Boston (Lincs.) Union. **C. Gordon Watson**, F.R.C.S., Junior Demonstrator of Anatomy at St. Bartholomew's Hospital. **F. E. A. Webb**, M.R.C.S., L.R.C.P.Lond., Certifying Factory Surgeon for the Cambridge District. **J. F. Wilkinson**, M.D., Ch.B.Melb., Physician to Out-paients, Melbourne Hospital.

**"The Hospital" Institutional Library.**

"Hospitals and Asylums of the World." 4 Vols., with a Portfolio of Plans, complete £12 12s.

"Burdett's Hospitals and Charities, 1902." 5s.

"Burdett's Official Nursing Directory, 1903." 3s. net.

"Cottage Hospitals, General, Fever, and Convalescent." 10s. 6d.

"Uniform System of Accounts for Hospitals and Public Institutions." New Edition. 4s. net.

"Hospital Expenditure: The Commissariat." 2s. 6d.

"A Legal Handbook for the Use of Hospital Authorities." 2s. 6d.

"The Cottage Hospital Case Book or Register of Patients." 10s. and 12s. 6d.

"The Furnishing and Appliances of a Cottage." 6d.

All these are published by the SCIENTIFIC PRESS, Ltd., and may be obtained through any bookseller, or direct from the publishers, 28 and 29 Southampton Street, Strand, London, W.C.

"Valuable little handbook."

*Nursing Notes.*

THE BURDETT SERIES.

"Can be strongly recommended to nurses."

*Treatment.*

### THE BURDETT SERIES OF POPULAR TEXT-BOOKS on NURSING.

Each Volume contains about 100 pages written by a competent authority on the subject treated.

Small Crown 8vo. Bound in cloth boards. Price 1/- each post free; or the Series complete 10/- post free.

No. 1.—**PRACTICAL HINTS ON DISTRICT NURSING.** By Miss Amy Hughes.

No. 2.—**HOSPITAL AND PRIVATE NURSING.** By Miss S. E. Orme.

No. 3.—**THE MIDWIVES' POCKET-BOOK.** With Prefatory Chapter on the Midwives' Bill. By Miss Honnor Morten, Author of "The Nurse's Dictionary," &c., &c.

No. 4.—**FEVERS AND INFECTIOUS DISEASES:** their Nursing and Practical Management. By William Harding, M.D.Ed., M.R.C.P.Lond., Author of "Mental Nursing," &c.

No. 5.—**NOTES ON PHARMACY AND DISPENSING FOR NURSES.** By C. J. S. Thompson.

No. 7.—**THE CARE OF CONSUMPTIVES.** By W. H. Daw, M.R.C.S., L.R.C.P.Lond.

No. 8.—**HOME NURSING OF SICK CHILDREN.** By J. D. E. Mortimer, M.B., F.R.C.S., L.S.A.

No. 9.—**MENTAL NURSING.** By William Harding, M.D.Ed., M.R.C.P.Lond.

No. 10.—**SICK NURSING AT HOME.** By Miss L. G. Moberly.

No. 11.—**GYNÆCOLOGICAL NURSING.** By G. A. Hawkins-Ambler, F.R.C.S., Author of "The Commonwealth of the Body."

No. 12.—**SYLLABUS OF LECTURES TO NURSES.** By Andrew Davidson, M.D.

*To be obtained of all Booksellers, or of*

The SCIENTIFIC PRESS, LTD., 28 & 29 Southampton Street, Strand, London, W.C.

